# Biological activities of optimized biosynthesized selenium nanoparticles using *Proteus mirabilis* PQ350419 alone or combined with chitosan and ampicillin against common multidrug-resistant bacteria

**DOI:** 10.1186/s12934-025-02783-0

**Published:** 2025-07-05

**Authors:** Laila A. Elshikiby, Zakaria A.M. Baka, Mohamed M. El-Zahed

**Affiliations:** Department of Botany and Microbiology, Faculty of Science, Damietta University, New Damietta, 34517 Egypt

## Abstract

**Background:**

One of the most common issues in the world is bacterial resistance and biofilms, which can prolong the healing period and the need for self-medication. Additionally, they may be linked to unsuccessful therapies, which raises death rates, healthcare expenses, and the need for additional hospitalization. Therefore, to protect the environment and improve human health, there is a need for the creative synthesis of novel antibacterial materials. *Proteus mirabilis* strain PQ350419 was isolated, identified, and utilized as an efficient bio-nano-factory for biosynthesizing selenium nanoparticles (Se NPs) and optimizing procedures. This study showcases a simple and cost-effective approach for green-synthesizing a selenium/chitosan/ampicillin nanocomposite (Se/CS/AMP) as a novel antibacterial and antibiofilm agent. Several analyses, such as transmission electron microscopy (TEM), X-ray diffraction (XRD), Fourier-transform infrared (FTIR) spectroscopy, zeta analysis, and ultraviolet-visible (UV-Vis) spectroscopy, were utilized to confirm and characterize the production of Se NPs and Se/CS/AMP. The absorption peaks for Se NPs and Se/CS/AMP were identified to be between 350 and 360 nm. The XRD data revealed the crystalline composition of the Se NPs loaded with CS and AMP. The FTIR spectra confirmed the presence of proteins that act as supporting and binding agents during synthesis. The stability of the prepared nanomaterials is improved by a strong negative surface charge of − 24.27 mV for Se NPs and − 23.92 mV for Se/CS/AMP. The particle sizes of Se NPs and Se/CS/AMP are shown by TEM to be in the ranges of 88–98 nm and 86–129 nm, respectively. Se NPs, either alone or in combination with chitosan (CS) and ampicillin (AMP), exhibited strong antibacterial activity against methicillin-resistant *Staphylococcus aureus* ATCC 43,300, *Bacillus cereus* ATCC 14,579, *Klebsiella pneumoniae* ATCC 11,296, and *P. mirabilis* PQ350419 in a dose-dependent manner. Compared to Se NPs and the common antibiotic AMP, the Se/CS/AMP combination demonstrated superior antibacterial activity. In comparison to Se NPs (40, 70, 110, and 150 µg/ml, respectively), the nanocomposite produced MIC values of 30, 40, 60, and 100 µg/ml against *B. cereus*, *S. aureus*, *K. pneumoniae*, and *P. mirabilis*. When compared to untreated cells, treated cells exhibited significant morphological changes and deformities, such as cell wall distortion, the separation of the cell wall from the plasma membrane, the formation of vacuoles, and complete cell lysis, according to TEM ultrastructure studies of bacteria treated with nanocomposite. Se/CS/AMP at 100 µg/ml was sufficient to prevent biofilm formation by up to 50% in *S. aureus*, *K. pneumoniae*, and *P. mirabilis*. The cell viability of the Vero cell line was significantly reduced (*p*˂0.05) in the cytotoxicity test of Se NPs alone at a concentration of 40.95 ± 2.34 µg/ml, and in its nanocomposite at a concentration of 199.09 ± 2.61 µg/ml. This indicates the nanocomposite’s safety by showing its minimal harmful impact on the Vero cell line.

**Conclusion:**

Se/CS/AMP has revealed an antibacterial and antibiofilm agent that could be useful in various industrial, medicinal, and environmental applications. This study introduces a work that presents an alternative, safe, promising, and efficient nanocomposite for treating harmful bacteria in humans and animals. This treatment is based on the synergistic effectiveness of Se NPs, CS, and AMP.

## Background

Antimicrobial resistance (AMR) can be defined as the defense mechanisms that microbes have developed against the effects of antibiotics or drugs [1]. Several microorganisms, especially bacteria, have the ability to develop resistance to the point where one or more antimicrobial agents are no longer effective. There are various bacterial resistance mechanisms against available antibiotics, such as limiting drug uptake, modifying drug targets [2], inactivating drugs, active drug efflux [3], and ribosome recycling and splitting [4]. Michael et al. [5] reported that antimicrobial resistance (AMR) was linked to approximately 3.57 million of the 4.95 million deaths that occurred globally. The UN and WHO estimate that the global impact of AMR is much greater than the 700,000 annual deaths currently, and could result in 10 million deaths annually by 2050 [6].

*Proteus* sp. is a genus of Gram-negative bacteria. It is an aerobic, motile, rod-shaped and multidrug-resistant bacterium that can travel across surfaces due to its swarming behavior, especially when the temperature is between 20 °C and 37 °C. There are three species of opportunistic human pathogenic *Proteus* sp., which include *P. vulgaris*, *P. mirabilis*, and *P. penneri* [7]. *P. mirabilis* is a bacterium that is commonly found in the urinary and digestive systems, but it can also cause infections in other parts of the body [8]. Common symptoms include infections of the urinary tract, wounds, respiratory system, and stomach [9]. Approximately 48% of *P. mirabilis* strains exhibit antibiotic resistance and are classified as multidrug-resistant or extended-spectrum beta-lactam bacteria [10].

Nowadays, nanomaterials are commonly used individually or combined with antibiotics as promising and effective antimicrobial agents to combat AMR problems [11] – [12]. Nanoparticles (NPs) range in size from 1 to 100 nm and offer the following benefits: low toxicity, minimal pathogen resistance, chemical stability, and potential antibacterial properties [13] – [14]. Two methods have been established to describe the various options for creating nanostructures. These manufacturing approaches fall under two categories: top-down and bottom-up, which differ in quality, speed, and cost. NPs can be synthesized in many ways, including physical, chemical, or biological methods. The rising costs and harmful effects of physical and chemical methods have prompted a shift towards green production of NPs. As a result, scientists are now turning to biological molecules like microbes [15], biomolecules, plants, and plant extracts as alternative reducing agents in order to find more cost-effective solutions. Bacteria can be cultivated in artificial settings with an ideal growth rate, making them a more feasible option for NP synthesis than other microorganisms. Inorganic NPs can be produced by bacteria either extracellularly or intracellularly. Due to simpler low-flow and purification processes, the synthesis of extracellular NPs is preferred over the intracellular approach. Several reports have documented the ability of various bacterial species to utilize detoxification processes to produce NPs, such as *Bacillus* sp., *Staphylococcus* sp., *Microbacterium* sp., *Rhodospirillum* sp., *Acinetobacter* sp., *Klebsiella* sp., and *Pseudomonas* sp [16–19].

NPs such as Ag, Cu, Au, Fe, and Se are essential in various fields, including medicine, industry, food, agriculture, and environmental applications [20] – [21]. Selenium NPs (Se NPs) were selected over other NPs due to their numerous benefits, including their crucial role in improving human health through functions in antioxidant defense, selenium-proteins, immunological regulation, and other physiological processes [22]. Furthermore, Se NPs exhibit a number of advantages, including degradability, low cytotoxicity, bioavailability, anti-inflammatory, antioxidant, antiviral, antimicrobial, and anticancer potential. This makes them one of the best choices for clinical use and exceptional in nanotherapy [23, 24].

Antimicrobial NPs can cause microbial cell death through various modes of action, including microbial cell membrane disruption, production of reactive oxygen species (ROS), suppression of spore germination, and protein regulation [25, 26]. On the other hand, some bacteria can resist NPs due to changes in environmental conditions such as pH, salts, shape, size, and chemistry of NPs, as well as the external medium [27]. The interactions between microbes and NPs can be affected by even minor adjustments to these factors or environments. Active efflux, biofilms, volatilization, enzyme detoxification, and genetic alterations have all been used to demonstrate how microbes have adapted to NPs. *E. coli* CCM 3954, *Escherichia coli* 013, and *P. aeruginosa* CCM 3955 become resistant to Ag NPs following repeated treatment and exposure [28].

Nanocomposites (NCs) are emerging as the materials of the future and offer new alternatives to the current limitations of monolithic NPs. Improved mechanical properties and a high surface-to-volume ratio, which allow for better filler dispersion and smaller filler sizes, are the main advantages of NCs over other composite materials.

High ductility without sacrificing strength [29]. According to their matrix components, NCs can be categorized as metal matrix NCs (MMNC), polymer matrix NCs (PMNC), or ceramic matrix NCs (CMNC) [30]. Chitosan (CS) is a renewable natural polymer. It has many reactive functional groups, such as -OH and -NH_2_ [31]. Chitosan (CS) is widely recognized as a safe, hydrophilic, biocompatible, non-immunogenic, biodegradable, and economical polymer. As a result, it is currently widely used in various fields including biotechnology, food, medicine, industry, and agriculture [11]. Several investigations have documented that the surface modification of Se NPs greatly impacts their antimicrobial action when combined with polysaccharide compounds [32–34]. Therefore, the current study aimed to biosynthesize, optimize, and characterize a new nanocomposite (NC) consisting of Se NPs, CS, and ampicillin (AMP) and to test its antimicrobial activity multidrug multidrug-resistant bacterial strains.

## Materials and methods

### Sample selection and collection

Twenty-two samples (12 females and 10 males), including urine, stool, and surface swap samples, were collected from various private clinics and medical analytical laboratories in New Damietta City, Kafr Elbattikh, and Faraskour (Damietta Government, Egypt, 31.4°N 31.72°E) in April and May 2024. The samples were processed according to the protocols described by Santiago-Rodriguez et al. [35] and Zboromyrska & Vila [36]. The samples were chosen based on the following inclusion criteria: cooperative patients aged 20 to 50 years who had bacterial infections. The current study did not include patients with complicated systemic disorders, immune system impairments, physical limitations, or psychological problems. Samples were obtained either on the same day or one to two days following the initial visit. Some patients had received antibiotics within the previous three months. Samples were collected in sterile cups or glass bottles and promptly delivered to the Microbiology Laboratory, Faculty of Science, Damietta University, Egypt, in an icebox for further experiments. The review boards of the involved institutions reviewed and approved the study, and all patients or their parents or guardians provided their informed consent.

### Isolation and purification of drug-resistant pathogenic bacteria

The isolation protocol for drug-resistant pathogenic bacteria involved using the spread plate method on a blood agar base (Oxoid Ltd., England) supplemented with 5% (wt/v) defibrinated, sterile sheep blood and 2 mg/l ceftazidime (MCKC) [37]. Each plate was then incubated for 24 h. at 37 °C, and the bacterial hemolytic reaction was recorded. To transfer and subculture each bacterial colony, MacConkey agar (MAC, Oxoid Ltd., England) and Cystine Lactose Electrolyte Deficient (CLED, Oxoid Ltd., England) agar plates were used at 37 °C for 48 h. Next, colonial morphological characteristics and color change of colonies and media were recorded. Isolates of *P. mirabilis* were selected, purified, and subjected to species-level identification using the Vitek 2 system (BioMerieux, Marcy-l’Étoile, France) [38]. The bacterial isolates were then preserved in agar slants for further investigations.

### Antibiotic sensitivity test (AST)

The AST of bacterial isolates was conducted using the disc diffusion technique [39]. Specifically, 50 µl of each bacterial suspension (0.5 MacFarland Standard, 1.5 × 10^8^ CFU/ml) was inoculated separately into Mueller-Hinton agar (MHA, Oxoid Ltd., England) flasks, which were then poured into sterile Petri dishes. After solidification, different antibiotic discs from different classes were added aseptically: amoxicillin/clavulanate (20/10 µg/ml), ampicillin (30 µg/ml), cefotaxime (30 µg/ml), ceftazidime (30 µg/ml), chloramphenicol (30 µg/ml), doxycycline (30 µg/ml), levofloxacin (5 µg/ml), nalidixic acid (30 µg/ml), and vanomycin (30 µg/ml). The diameter of the zones of inhibition (ZOI) was recorded and measured (mm) after incubation at 37 °C for 24 h.

### Detection of biofilm formation

The biofilm formation of *P. mirabilis* isolates was investigated using a crystal violet-based assay [40]. 0.5 MacFarland bacterial suspension from *P. mirabilis* isolate was prepared in tryptic soy broth (TSB, Oxoid Ltd., England). Polystyrene titer plates with 96 wells were filled with aliquots of 20 µl of the prepared suspension. Subsequently, 180 µl of TSB was added to the wells, and the culture was cultured for 24 h. at 37 °C. After the incubation, cultures were discarded from the wells, and plates were air-dried at 37 °C. After washing the plates with sterile distilled water, the wells were filled with methanol and shaken well at 400 rpm for 20 min. The solutions were then discarded from the wells, and the plates were air-dried at 37 °C. Next, the wells were filled with 0.1% crystal violet solution and shaken at 400 rpm for 10 min. The crystal violet was then discarded from the wells by rinsing them thoroughly with water. Then, plates were allowed to dry at 37 °C for 20 min. Wells were filled with methanol and shaken for 4 min. Absorbance at 570 nm was determined using spectrophotometry. The following equation was used to calculate the quantitative evaluation of biofilm formation: T = X_nc_ + 3δ, where T represents the threshold absorbance, X_nc_ is the arithmetic mean of the negative control, and 3δ is three times its standard deviation. The absorbance of biofilm was defined as a value below the total sum. If the sum value of a biofilm fell between T and 2T, it was considered mild; if it fell between 2T and 4T, it was considered moderate; and if it exceeded 4T, it was considered strong.

### Biosynthesis of Se NPs

In 100 ml nutrient broth flasks, a 0.5 MacFarland standard was produced from each bacterial isolate. The bacterial metabolites were collected by centrifugation at 5000 rpm for 15 min, filtered using a 0.22 μm syringe filter (Millex GV, Millipore), and then transferred to a fresh flask. After preparing a 3 mM Na_2_SeO_3_ solution, it was combined with the cell-free bacterial metabolite in a 1:1 (v/v %) ratio. The flasks were then shaken at 150 rpm and 37 °C until the solution turned red. The production rate of Se NPs was measured using a double-beam spectrum UV–Vis spectrophotometer V-760 (JASCO, UK) [41].

### Molecular characterization and identification of multidrug-resistant *proteus mirabilis* AUF1

Bacterial cells were collected from a 10 ml overnight culture of nutrient broth medium (Oxoid Ltd., England) using centrifugation at 8000 rpm for 10 min. They were then washed with TE buffer (10 mM Tris chloride, 1 mM EDTA, pH 8.0) and resuspended in 200 µl of TE buffer. Chromosomal DNA extraction was carried out following the instructions of the QIAamp DNA mini kit. The resulting chromosomal DNA (100 µl) was stored in a -20℃ freezer for PCR and sequence analysis [42].

The PCR was performed to amplify a 16S rRNA using the universal primers F27 and R1492. The cycling conditions of the PCR were adjusted with early denaturation at 94 °C for 15 min, followed by 35 cycles of subsequent denaturation at 94 °C for 30 s. Primers were annealed for 1 min at 56 °C, extended for 1 min at 72 °C, and then further extended for 10 min at 72 °C [43]. The samples were subjected to agarose gel electrophoresis following the protocol outlined by Sambrook et al. [44]. After approximately 30 min, the run was stopped, and the gel was transferred to the UV transilluminator.

An Applied Biosystems 3130 automated DNA Sequencer was used to sequence a purified PCR result in both forward and reverse orientations. First, a BLAST analysis was performed to determine sequence identity to GenBank accessions [45, 46]. The CLUSTAL W multiple sequence alignment program, version 12.1 of the MegAlign module of Lasergene DNAStar software Pairwise (Madison, Wisconsin, USA), developed by Thompson et al. [47], was used to compare the sequences. MEGA6 was used for phylogenetic analyses, employing which used maximum likelihood, neighbor joining, and maximum parsimony methods.

### Optimization conditions for Se NPs biosynthesis

The optimal conditions for biogenic production of Se NPs were determined by testing a range of concentrations from Na_2_SeO_3_ (0–50 mM), ratios between cell-free bacterial metabolites and Na_2_SeO_3_ (1:1–1:10 v/v %), incubation periods (12–60 h.), pH levels (4–10), and temperatures (10–50 ^o^C). A double-beam UV–Vis spectrophotometer V-760 was used to assess the concentration and production rate of Se NPs [48].

### Green synthesis of selenium/chitosan/ampicillin nanocomposite (Se/CS/AMP)

60 mg of chitosan (deacetylation degree: ≥85%, Sigma-Aldrich, USA) was dissolved and stirred in 1% aqueous acetic acid (40 ml) until completely dissolution. After complete dissolution, 1 ml Na_2_SeO_3_ (5 mM), 1 ml of bacterial metabolite, and 60 mg of AMP were added to a sodium tripolyphosphate (STPP) solution and stirred for about 10 min at room temperature (25 °C). The solution of Se/CS/AMP was then centrifuged, dried, and stored for future experiments [49]. The drug loading of the Se/CS/AMP was calculated as follows: (weight of AMP loaded into the Se/CS/AMP) / (total weight of Se/CS/AMP).

### Characterization of the prepared nanomaterials

The FTIR (FT/IR-4000 Series, JASCO, UK), X-ray diffractometer (XRD, LabX XRD-6000, Shimadzu, Japan), TEM (JEM-2100, JEOL, Japan), and Nano-ZS90 Zetasizer (Malvern, UK) were used to study and analyze the characteristics of the optimized Se NPs and Se/CS/AMP.

### Antibacterial action of se/cs/amp

The antibacterial efficacy of Se/CS/AMPs was assessed against Gram-negative bacteria (*P. mirabilis* PQ350419 and *K. pneumoniae* ATCC 11296) and Gram-positive bacteria (*B. cereus* ATCC 14579, methicillin-resistant *S. aureus* ATCC 43300) in comparison to Se NPs and AMP using the agar well diffusion method [50]. Each bacterial strain was inoculated into MHA plates. Different concentrations (50, 100, and 150 µg/ml) of each tested antibacterial agent were prepared in Dimethylformamide (DMF), added into wells prepared on MHA plates, and then incubated at 37 °C for 48 h. The resulting zones of inhibition (ZOI) were measured in millimeters (mm). to calculate the fold increase in area for Se NPs combined with CS and AMP, the equation *(B*^*2*^*-A*^*2*^*)/A2* was used, where *A* represents the ZOI of Se NPs alone and *B* represents the ZOI of Se NPs combined with CS and AMP [51].

### Minimum inhibitory concentration (MIC)

The MIC values for Se NPs and Se/CS/AMP against the investigated bacterial strains were determined using Mueller-Hinton broth (MHB) medium following the broth dilution method [52]. To start, 0.5 MacFarland of each bacterial strain was added to MHB along with varying quantities (0–150 µg/ml) of Se NPs and Se/CS/AMP, then incubated at 150 rpm and 37 °C. After 24 h., the MIC values of Se NPs and Se/CS/AMP were measured spectrophotometrically at 600 nm.

### Minimum bactericidal concentration (MBC)

The pour plate method was used to seed 50 µl aliquots from each tube that did not demonstrate any apparent bacterial growth (MIC) on MHA plates. The plates were then incubated for 24 h. at 37 °C. Subsequently, bacterial colony growth was assessed, and MBC values were determined by enumerating the total colony-forming units per milliliter (CFU/ml) [53].

### Ultrastructural study

The antibacterial potential of Se NPs and Se/CS/AMP was studied by investigating their mechanism of action using *P. mirabilis* PQ350419 as a bacterial model. After treatment with MBC of each nanomaterial, MHB flasks were inoculated with a 0.5 MacFarland suspension of *P. mirabilis* PQ350419 and then incubated for 24 h. at 37 °C and 150 rpm. Se NPs and Se/CS/AMP were added, and the bacteria were allowed to proliferate for 2 h. Cells were obtained through centrifugation at 8000 rpm for 20 min in an aseptic environment. Following this, the cells were cleaned using sterile distilled water as part of a routine procedure. Bacterial cells were then fixed with 2.5% glutaraldehyde in 0.1 M cacodylate buffer at pH 7 for 20 min before extraction. Once the preserved cells were dried with a series of ethanol, samples were double-stained and examined using a TEM on carbon-coated copper grids (Type G 200, USA) [54].

### Antibiofilm test

Se NPs and Se/CS/AMP were tested for their antibiofilm potential against *S. aureus* ATCC 43,300, *K. pneumoniae* ATCC 11,296, and *P. mirabilis* PQ350419. Aliquots of 10 µl of bacterial suspension (0.5 MacFarland) were inoculated into wells of 96-well polystyrene titer plates containing 100 µl of glucose-TSB (1% w/v). Se NPs and Se/CS/AMP (50, 100, and 150 µg/ml) were added separately to the wells and then incubated for 24 h. at 37 °C. Controls, media containing bacterial suspension in the positive control well, and sterile medium alone in the negative control well were also prepared. After incubation, cultures were removed, and wells were cleaned with sterile distilled water. The remaining biofilm was stained with ethanol (95%) and crystal violet (0.25%). The absorbance of the adhered cells-dye mixture was measured spectrophotometrically at 570 nm. The following equation was used to estimate the biofilm inhibitory percentage: Antagonistic efficiency = (*A*_*1*_-*A*_*2*_)/*A*_*1*_ × 100, where *A*_*1*_ is the absorbance of the untreated control, and *A*_*2*_ is the absorbance of the treated samples [55].

### Cytotoxicity activity of Se NPs and se/CS/AMP

Vero cells (ATCC, Rockville, MD) were utilized to study the cytotoxic effect of Se NPs and Se/CS/AMP. The Vero cells were grown in Dulbecco’s Modified Eagle’s medium (DMEM) supplemented with 50 µg/ml gentamicin, 10% heat-inactivated fetal bovine serum, and 1% L-glutamine. Using a multichannel pipette, 96-well tissue culture plates were seeded with 1 × 10^4^ cells per well in 100 µl of growth medium during the exponential growth phase. The cells were then allowed to adhere for 24 h. Afterwards, Se NPs or Se/CS/AMP were applied to the corresponding wells to achieve final concentrations ranging from 0 to 500 µg/ml. The MTT assay, as outlined by Mosmann [56], was used to determine the number of viable cells after a 24-hrs. incubation at 37 °C in an incubator with 5% CO_2_. Plotting the relationship between Se NPs or Se/CS/AMP concentration and the remaining cells allowed for the generation of survival curves for each cell line following treatment with Se NPs or Se/CS/AMP.

### Statistical analysis

The data was statistically analyzed using SPSS version 18 software. A one-way analysis of variance (ANOVA) was utilized for the analysis, with each experimental value presented as the mean ± standard deviation (SD). Intergroup comparison was determined using either the least significant difference (LSD) test or Duncan’s multiple range test, with a significance level set at less than 0.05. Each experiment was conducted three times.

## Results and discussion

*P. mirabilis*, a multidrug-resistant Gram-negative enteric bacterium, has been identified as a significant cause of infections in both community and healthcare settings. These infections include those of the urinary tract, abdominal cavity, and bloodstream [57]. Various integron- and plasmid-mediated determinants of antibiotic resistance may be found in *P. mirabilis* as well as other members of the Enterobacteriaceae family [58]. The incidence of multidrug-resistant strains of *P. mirabilis* is relatively high in certain conditions. These strains typically produce the AmpC-type cephalosporinase or extended-spectrum β-lactamases (ESBLs), and infrequently, carbapenemases [59].

### Isolation, purification, and phenotypic characterization of *P. mirabilis* isolates

Among the 24-hrs. developed resistant bacterial colonies, 16 Gram-negative, rod-shaped bacteria were observed. These bacteria were positive for citrate and glucose fermentation, but negative for lactose fermentation and non-spore forming. They were selected for subculturing on MacConkey and CLED agar plates. These bacteria were differentiated on MacConkey agar plates, with 8 bacterial isolates showing a yellow color, 5 isolates appearing colorless, and 3 isolates displaying a pale pink hue. The selective effect of this medium is attributed to its components, including bile salts and crystal violet, which inhibit most Gram-positive bacteria while promoting the growth of Gram-negative bacteria, particularly those belonging to the Enterobacteriaceae family. Smooth, pale, or colorless colonies were chosen and cultured on CLED agar plates [60]. After 48 h., translucent blue colonies were selected and purified. Species-level identification was performed using the Vitek 2, which indicated the presence of 5 *P. mirabilis* isolates encoded with AUF1, AUF4, DUF, EUF18, and FUM2 (Table 1). All *P. mirabilis* isolates were screened for the production of Se NPs.

**Table 1:** Biochemical tests for *P. mirabilis* isolates

Biochemical test	AUF1	AUF4	DUF	EUF18	FUM2
Citrate utilization	+^*^	+	+	+	+
Catalase	+	+	+	+	+
Oxidase	-	-	-	-	-
Gas from glucose	+	+	+	+	+
Indole production	-	-	+	-	+
Methyl red	+	+	+	+	+
Voges-Proskauer	-	-	+	+	-
Urease	+	+	+	+	+
Nitrate reduction	+	+	+	+	+
H_2_S production	+	-	-	-	-
Lactose fermentation	-	-	-	-	-
Glucose fermentation	+	+	+	+	+
Mannitol fermentation	-	-	+	-	-

^*^+ = Present; - = Absent

### AST pattern for *P. mirabilis* isolates

Table 2 demonstrates the strain’s resistance to every antibiotic that has been tested. The results show that *P. mirabilis* isolates have a resistance percentage of 100% to ampicillin, amoxicillin/clavulanate, doxycycline, and ceftazidime. Additionally, isolates have a resistance percentage of 80 against levofloxacin and vancomycin and a percentage of 60 against cefotaxime. While chloramphenicol and nalidixic acid showed a resistance percentage of 20% against those isolates and sensitivity ratio values of 80%, the obtained results indicated multidrug resistance behavior for the *P. mirabilis* isolates. Alsaimary et al. [61] recorded the resistance of *Proteus* sp. isolates against ampicillin (75%), erythromycin (60%), and penicillin (75%). Other patterns of resistance ranged from 30 to 60% for various antibiotics such as vancomycin, tetracycline, ciprofloxacin, augmentin, gentamicin, and trimethoprim. Dawoud et al. [62] isolated various *P. mirabilis* clinical isolates and reported their highest resistance to ampicillin (92%) and amoxicillin-clavulanic acid (88%). However, the isolates exhibited the lowest resistance to imipenem (30.7%). The study also documented the presence of multidrug-resistant *P. mirabilis* isolates, accounting for 42.7% of the total.

**Table 2:** Patterns of antibiotic resistance and sensitivity in *P. mirabilis* isolates

Antibiotic	Concentration (µg/ml)	Antibiotics susceptibility	
		% Sensitivity	% Resistance
Amoxicillin/clavulanate	20/10	0^*^	100
Ampicillin	30	0	100
Cefotaxime	30	40	60
Ceftazidime	30	0	100
Chloramphenicol	30	80	20
Doxycycline	30	0	100
Levofloxacin	5	20	80
Nalidixic acid	30	80	20
Vanomycin	30	20	80

^*^numbers = Antibiotics susceptibility percentage; 0% Sensitivity or 100% Resistance = Absence of *P. mirabilis* isolates sensitivity

### Assessment of biofilm formation of *P. mirabilis* isolates

**Fig. 1:**
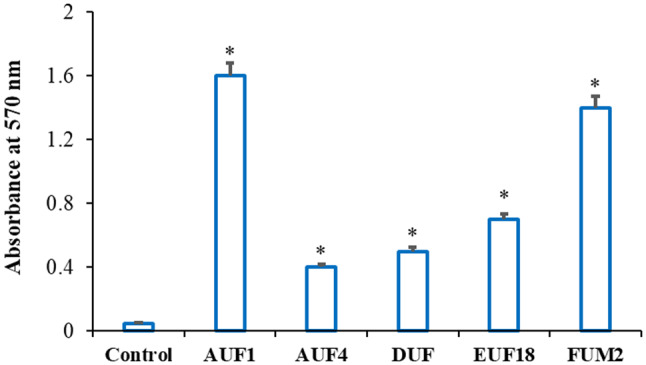
*P. mirabilis* isolates biofilm formation. * indicates considerably higher biofilms than those under control conditions (*P* < 0.05)

All tested isolates were found to be biofilm producers. Using the crystal violet-based method, two isolates, *P. mirabilis* AUF1 and FUM2, were identified as strong biofilm producers, while the remaining isolates were classified as weak biofilm producers (Fig. 1). Similarly, Wang et al. [63] used the crystal violet staining method to detect biofilm formation and observed the ability of two *P. mirabilis* strains isolated from clinical urine samples. Additionally, O’May et al. [64] found that *P. mirabilis* was able to form biofilms at 37 °C in polystyrene tubes within 24 h., with increased formation after 48 h.

### Synthesis of Se NPs by *P. mirabilis* isolates

**Fig. 2:**
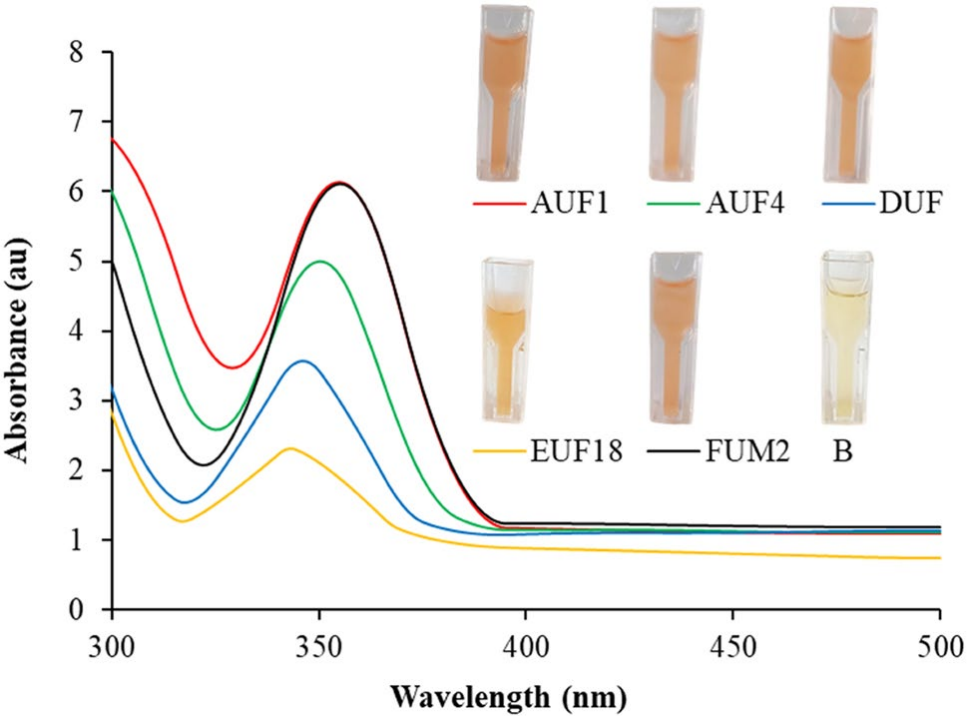
UV–Vis spectroscopy and change in color studies during the formation of Se NPs using *P. mirabilis* isolates. (**B**) The reaction mixture at the beginning of the Se NPs production

During the assessment of Se NPs generation by the *P. mirabilis* isolates, the color of the reaction mixture changed from pale yellow at the beginning of the experiment to red at the end of the incubation period (Fig. 2). The red hue of the sample was attributed to the activation of the Se NPs’ surface plasmon vibrations [65]. The color of the sample did not change in the control studies, indicating that only cell-free bacterial metabolites and proteins were responsible for producing Se NPs. UV–Vis spectrometric analysis confirmed the production of Se NPs, showing that *P. mirabilis* AUF1 and FUM2 had the highest production rates compared to other bacterial isolates. Production of Se NPs cell-free metabolites from different *P. mirabilis* isolates showed absorbance peaks ranging from 350 to 360 nm. Srivastava & Mukhopadhyay [66] biosynthesized Se NPs using the bacteria *Z. ramigera*, resulting in an absorption peak at 330 nm., Shakeri et al. [67], on the other hand, produced Se NPs using *P. aeruginosa*, which exhibited an absorption peak at 275 nm.

### Molecular characterization of *P. mirabilis* AUF1

**Fig. 3:**
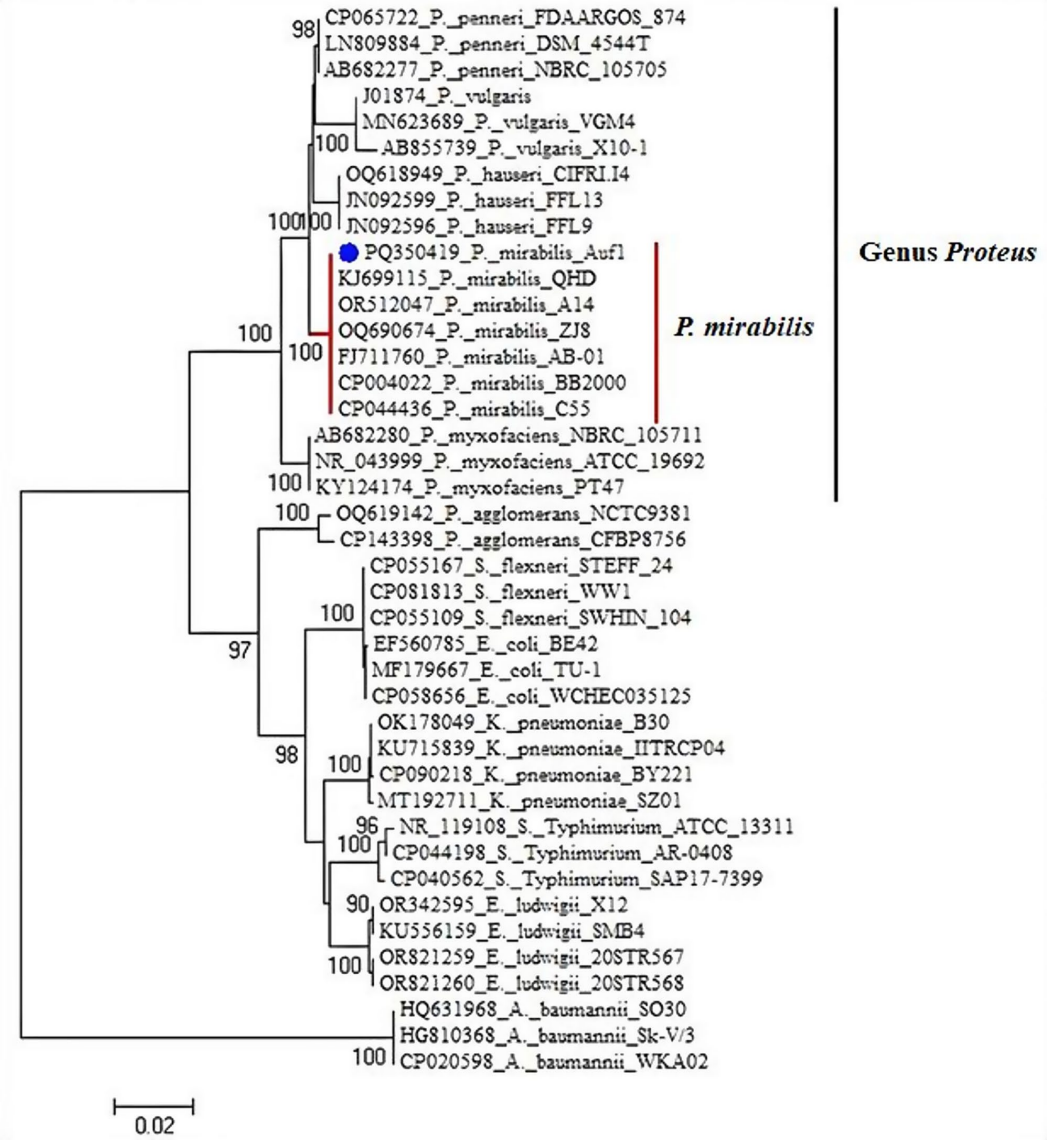
*P. mirabilis* AUF1 bacterial strain’s 16S rRNA sequences were used to create the phylogenetic tree. A bootstrap of 100 replicates was used to determine the number of branch nodes. Bootstrap values greater than 50% are shown. The strain of the genus *Proteus* was employed as an out-group

For the generation of Se NPs, the *P. mirabilis* AUF1 isolate was chosen as a potent bio-nanofactory. The identity of the *P. mirabilis* AUF1 isolate was confirmed using 16S rRNA sequencing. Following the sequencing of the AUF1 isolate’s 16S rRNA gene, BLAST analysis, which compares sequences from the NCBI database, revealed a connection between the strain and the Enterobacteriaceae family. The genus *Proteus* was associated with the AUF1 strain, and in fact, strain AUF1 and the strain of *P. mirabilis* were found to be 100% identical (Fig. 3). The partial 16S rDNA sequence of *P. mirabilis* AUF1 was added to GenBank under accession number PQ350419.

### Optimization of Se NPs production using *P. mirabilis* PQ350419

**Fig. 4:**
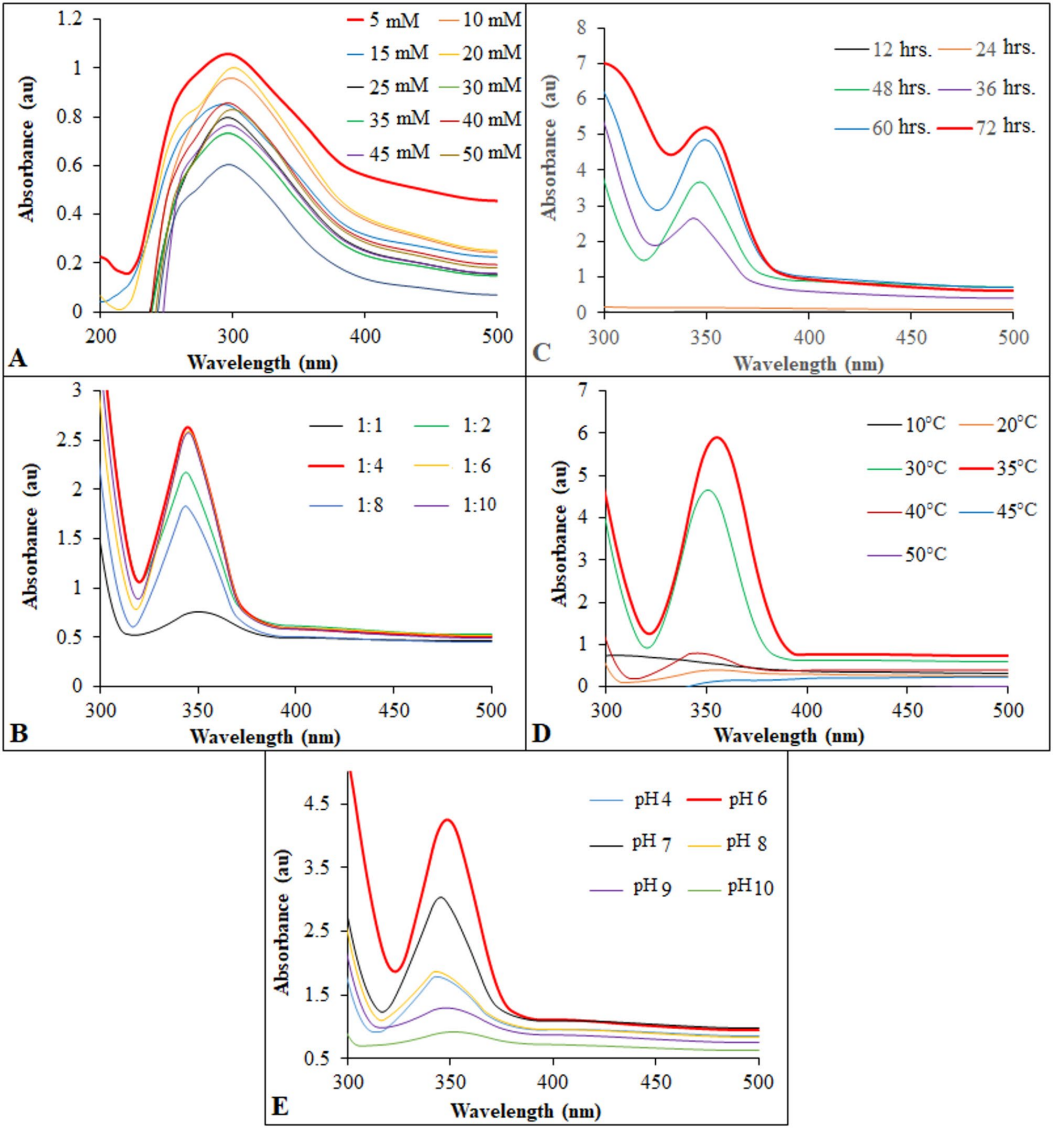
Optimization of Se NPs production using *P. mirabilis* PQ350419. (**A**) Concentrations (5–10 mM) of Na_2_SeO_3_. (**B**) Effect of different mixing ratio between cell-free bacterial metabolites and Na_2_SeO_3_ (1:1–1:10 v/v%). (**C**) Different incubation periods through Se NPs biosynthesis. (**D**) Effect of temperature (10–50 °C). (**E**) Effect of different pH value on Se NPs formation

The ratio of cell-free bacterial metabolites to Na_2_SeO_3_, temperature, pH, Na_2_SeO_3_ concentrations, and incubation time were investigated as variables for optimizing the biosynthesis of Se NPs (Fig. 4). Initially, different amounts of Na2SeO_3_ ranging from 5 to 50 µg/ml were added to the supernatant to determine the optimal selenite concentration (Fig. 4A). *P. mirabilis* PQ350419 exhibited the best results for high Se NPs concentration at 5 µg/ml of Na2SeO_3_, resulting a noticeable reddish color colloidal solution, indicating the reduction of selenite anions into reddish elemental Se NPs. By increasing the concentration of Na_2_SeO_3_ from 10 to 50 µg/ml, the intensity of the red color in Se NPs generation decreases. At lower concentrations, the production was minimal and slow, possibly due to lower levels of enzyme substrate. Conversely, higher concentrations (40–50 µg/ml) had a detrimental effect on Se NPs biosynthesis due to toxicity. These results are similar to those of Mollania et al. [68], who observed maximum absorbance for Se NPs production using *Enterobacter* sp. strain at a concentration of 4 µg/ml of selenite.

It is also nearly similar to the study of Mohamed & El-Zahed [12], where the red color intensity of Se NPs generation increases as the quantity of Na2SeO_3_ is increased from 1 to 5 µg/ml. The best and most ideal circumstances for producing Se NPs were found when 5 µg/ml of Na2SeO_3_ was mixed with cell-free bacterial metabolites in a 1:4 (v/v%) ratio (Fig. 4B). It was discovered that extending the incubation period, led to an increase in the production of Se NPs (Fig. 4C). The best and ideal production was observed after 36 h., continuing to increase until 72 h. Maximum synthesis of Se NPs occurred at 35 °C, with synthesis taking place between 30 and 40 °C (Fig. 4D). Our results align with those of Lortie et al. [69], who also found that Se NPs could not be effectively synthesized at temperatures above 35 °C. According to the earlier work, NPs aggregate into rods when the temperature increases from 80 °C to 100 °C.

The biomolecules and proteins that reduce Na_2_SeO_3_ are only active at temperatures of 30 °C and 37 °C, which may explain why 35 °C initially considered the ideal temperature. *C. reticulata* peel extract has been reported to be effective at 40 °C for the synthesis of Se NPs [70]. Additionally, the initial pH of the supernatant was monitored during the synthesis of Se NPs. The transition from pale yellow to red in the hue of Se solutions would indicate selenite reduction and the formation of Se NPs in different sizes and shapes. Therefore, pH would control the observation of different hues (Fig. 4E). Indeed, selenite ions and various functional groups, including carboxylate, hydroxyl, amine, and phosphoryl groups, on the biomass surface of polysaccharides, lipids, and proteins, have important physicochemical interactions [48, 71, 72]. Consequently, one factor that could have a significant impact on the biosorption process is the pH of the supernatant. Results indicated that the greatest amount of anionic selenite was absorbed at pH 6. At very basic and acidic pH values, red hue of the solution diminished and UV-Vis spectroscopy did not reveal any analytical evidence of nanoparticle formation. Additionally, El-Dein et al. [73] reported that bacterial formation of NPs was more efficient at pH 5–6 and the production rate increased with higher pH value until reaching pH 7, which produced stable NPs. Meanwhile, Mollania et al. [68] found that the maximum absorption of Se NPs occurred at a neutral pH.

### Characterization of Se NPs and se/CS/AMP

**Fig. 5:**
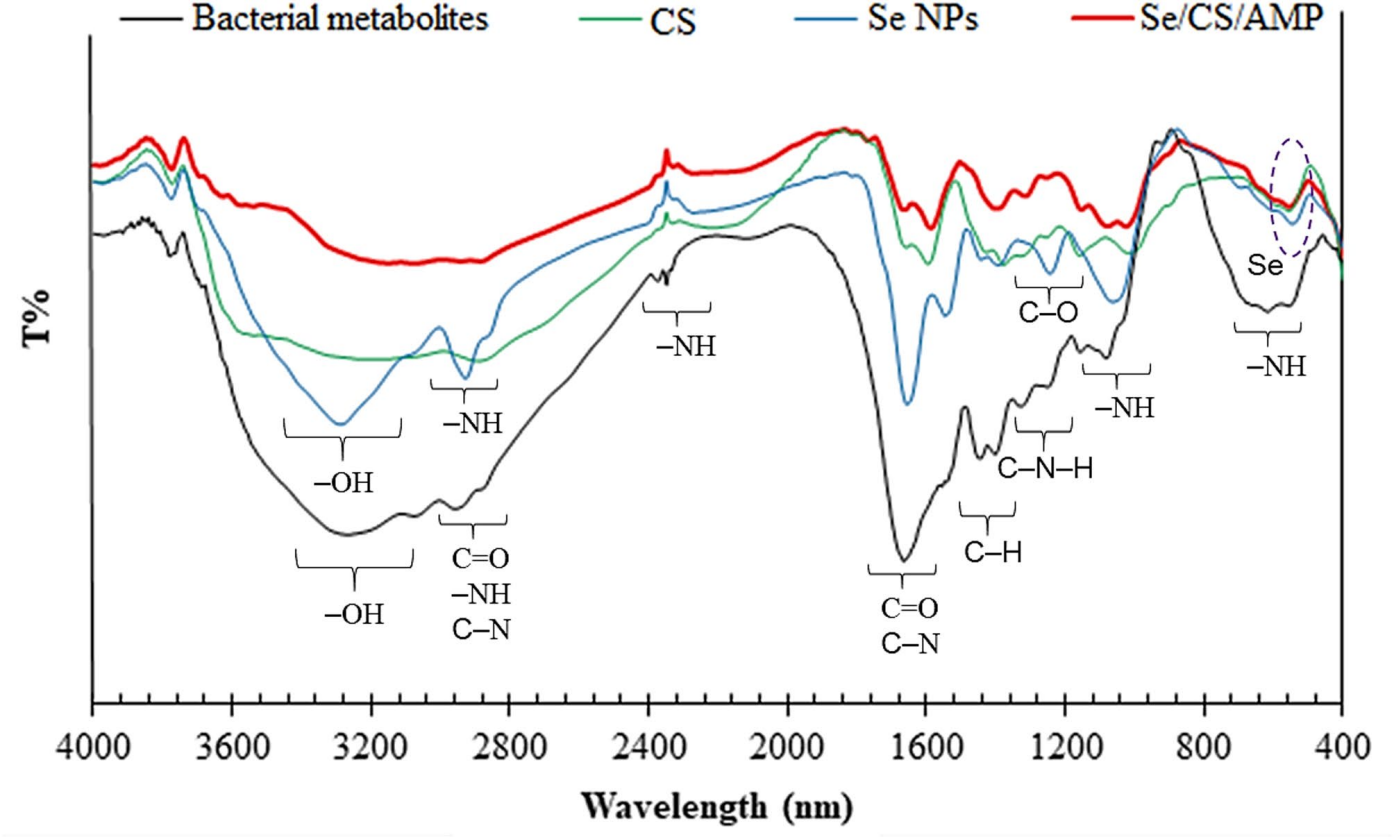
FTIR spectra of cell-free *P. mirabilis* PQ350419 metabolites, CS, Se NPs, and Se/CS/AMP

Biosynthesized Se NPs produced by *P. mirabilis* PQ350419 and Se/CS/AMP were characterized using FTIR, XRD, TEM, and zeta potential analyses. The bacterial crude protein and the protein linked to the surface of Se NPs displayed clear alterations in shape and peak location in their FTIR spectra (Fig. 5), indicating modifications in the secondary structure of the protein following NP formation. The interaction with Se NPs has been shown to impact the secondary structure of proteins, as indicated by FTIR data. Analysis of the Se NPs’ FTIR spectra revealed several protein-specific peaks at 544.792, 693.284, 1063.55, and 1243.86 cm^− 1^. It is hypothesized that NPs interacting with molecules containing these functional groups may experience increased stability as the binding proteins act as capping agents on the Se NPs [74].

The strong broad peaks at 3538.74 cm^− 1^ can be assigned to the hydroxyl (-OH) group. The peak at 1545.67 cm^− 1^ is attributed to C-H vibration. The C-O stretching mode may be responsible for the bands at 1397.17 cm^− 1^. Additionally, the amide I band is mainly a C = O stretching mode, the amide II band is a combination of N-H in-plane bending and C-N stretching that appears at bands 3000–2800 cm^− 1^, and the more complicated amide III band can be found close to 1397 cm^− 1^. The reduction production and stability of the metal ions brought about by the presence of enzymes indicates that molecules containing these functional groups are connected to the NPs. Metal NPs appeared at 400–700 cm^− 1^ confirming the successful formation of Se NPs [75]. The Se vibration (C–Se) accounts for the emergence of 525 cm^− 1^ (AMP/CS/SeNC) and 539 cm^− 1^ (Se NPs). Our findings were validated by Qian et al. [76] and El-Fawal et al. [77], who demonstrated that the Se-Se vibration is responsible for the peak at 493 cm^− 1^ and 606 cm^− 1^, respectively. There are further Se-related bonds found at C-Se (606 cm^− 1^) in the FTIR spectra of cell-free *P. mirabilis* PQ350419 metabolites, CS, Se NPs, and Se/CS/AMP.

**Fig. 6:**
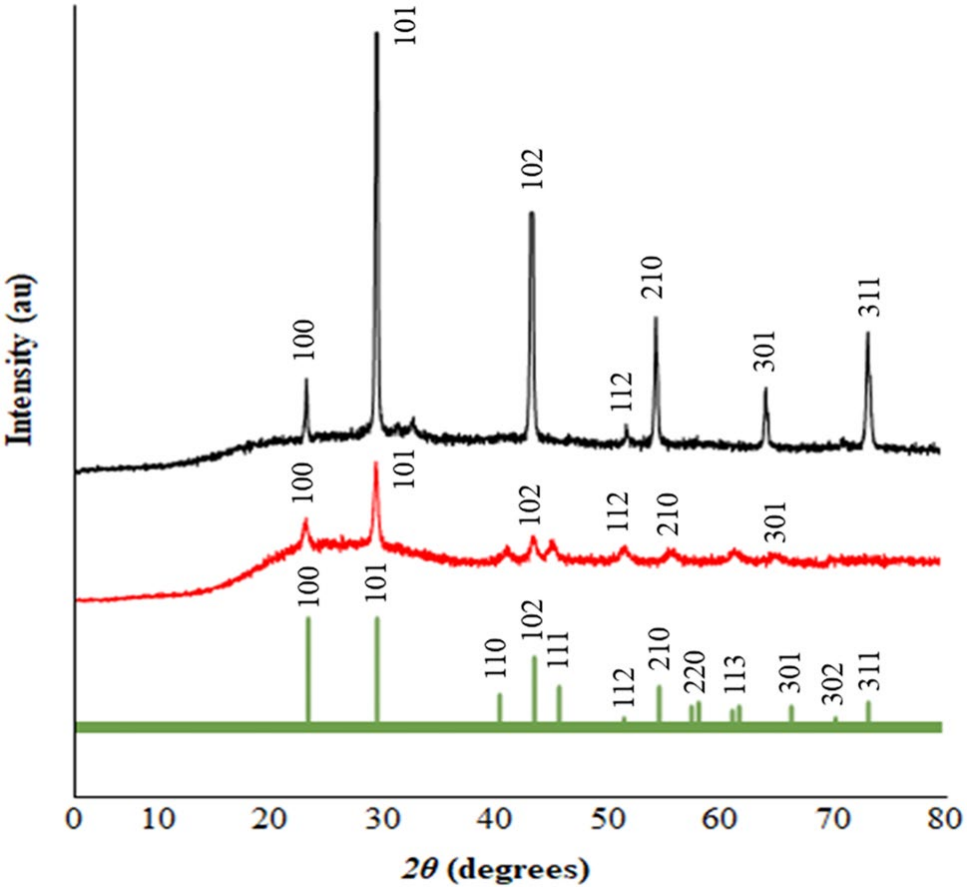
XRD patterns of Se NPs, and Se/CS/AMP

XRD can be used to reveal the existence of Se NPs and is often applied to analyze an object’s specific chemical configuration and crystal structure. The XRD patterns obtained for the Se NPs that were synthesized by *P. mirabilis* PQ350419, as well as AMP/CS/SeNC, are (Fig. 6).

The obtained data displayed distinctive peaks of Se NPs at angles 23.620°, 30.051°, 43.813°, 52.055°, 56.094°, 65.742°, and 73.011°, corresponding to crystallographic planes of (100), (101), (102), (112), (210), (301), and (311). This indicates that the precipitate was composed of pure crystalline Se, with lattice constants a and c less than 4Å. The obtained diffraction peaks suggest a spherical structure of Se NPs in agreement with the standard JCPDS data (JCPDS No. 06-0362).

The XRD results of AMP/CS/SeNC exhibited distinct characteristic peaks at angles 23.750°, 30.079°, 43.918°, 52.068°, 56.118°, and 65.429° corresponding to crystallographic planes of (100), (101), (102), (112), (210), and (301). This confirms the successful coupling between Se NPs with CS and AMP. The current data is nearly similar to the results documented by Srivastava & Mukhopadhyay (2013).

The lack of contaminant diffraction peaks suggests a pure final product. To determine the crystallite size of Se NPs, Scherrer’s equation was used: D = *Kλ*/ (*β cos θ*); where the particle size constant *K* = 0.94, x-ray wavelength *λ* = x-ray wavelength, *β* = full width at half maximum, and *θ* = diffraction angle. The average crystalline size of biosynthesized Se NPs was calculated to be 84.64 nm, while the size of AMP/CS/SeNC was 111.13 nm using Scherrer’s equation.

**Fig. 7:**
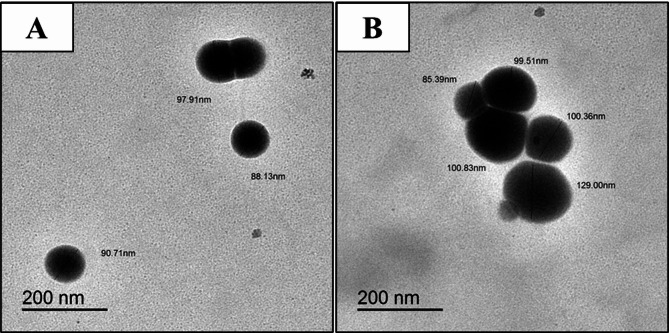
TEM micrographs of Se NPs; (**A**), and Se/CS/AMP; (**B**). Bar scales = 200 nm

An essential tool for assessing and studying the size and shape of NPs is the TEM investigation. The results of Scherrer equation are consistent with the TEM pictures, which clearly display the crystalline and evenly spilt NPs with diameters ranging from 88 to 98 nm for Se NPs and from 86 to 129 nm for Se/CS/AMP (Fig. 7). The TEM micrograph of Se NPs demonstrated their uniform distribution, spherical shape, and absence of aggregation. Se NPs produced by *S. parvulus* MAR4 ranged in size from 39.7 to 98.1 nm, while Se/CS ranged in size from 48.8 to 129 nm, as reported by Hassan et al. [72]. According to Asad Ullah et al. [78], the diameter of Se NPs produced by *B. subtilis* BSN313 was measured between 280 and 630 nm.

**Fig. 8:**
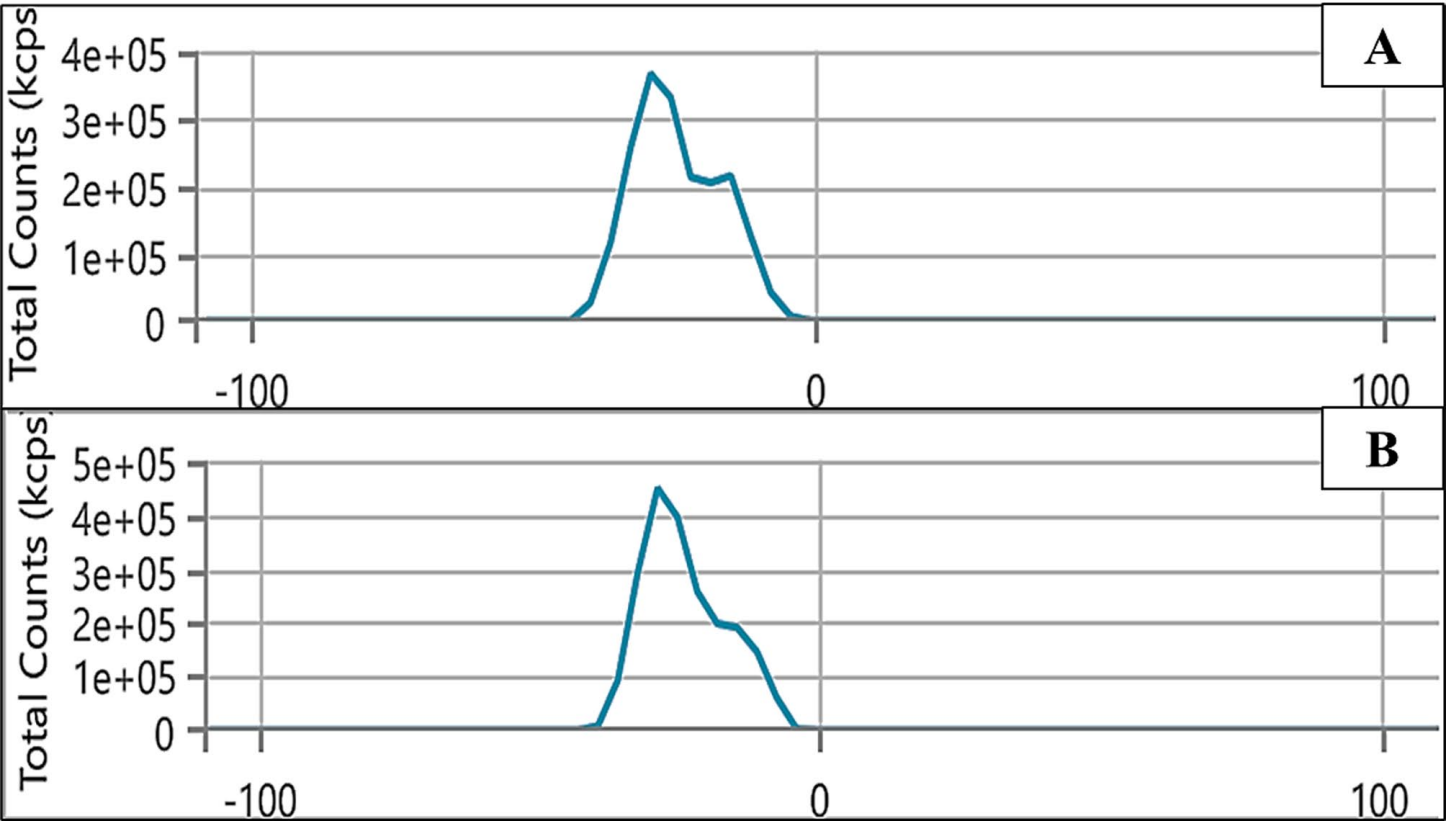
Zeta potential of Se NPs; (**A**), and Se/CS/AMP; (**B**)

One crucial measure of the stability of NPs’ colloidal dispersion is the zeta potential. The zeta potential is the measurement of an effective electric charge on the surface of NPs. NPs with a higher zeta potential experience greater electrostatic repulsion, making them more stable. The zeta potential measurements showed that the biosynthesized Se NPs had a negative charge (of -24.27 mV) while Se/CS/AMP had a zeta potential of -23.92 mV (Fig. 8). According to Laslo et al. [79], *L. casei* generated Se NPs with a maximum zeta potential of − 23 mV. However, as stated by Mohamed & El-Zahed [12], *L. fermentum* produced Se NPs with a zeta potential of -11.8 mV, indicating a negative charge. The negative sign of the zeta potential suggests that the overall charge of the object is negative. This negative zeta potential of Se/CS/AMP is mainly attributed to the presence of negatively charged binding proteins that act as capping agents on the NPs’ surface. CS, a polymer with a positive charge, can acquire a negative charge under specific conditions, either through deprotonation of its amine groups or by adsorbing negatively charged species.

Additionally, AMP, primarily a zwitterionic molecule, can also contribute to the overall negative charge depending on the pH and the surrounding environment [80]. The optimum pH value for Se NPs production was pH 6, which could lead to the presence of negatively charged AMP [81]. The stability of NPs is increased by electrostatic repulsion caused by the negative charges on the surface of basic groups.

**Fig. 9:**
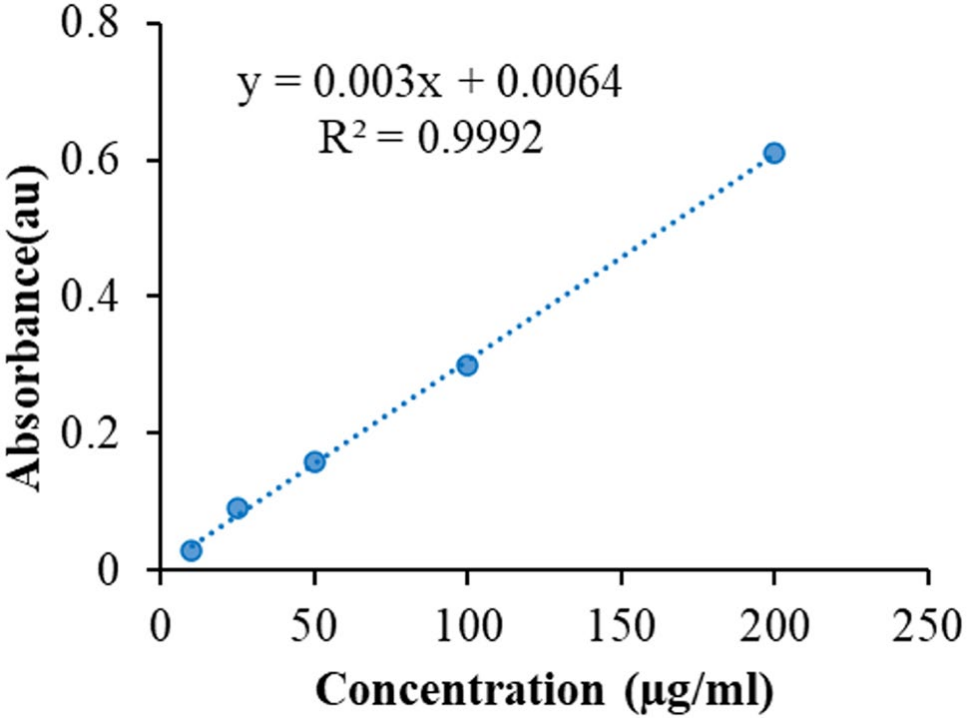
Standard curve of AMP in pure water

In order to estimate AMP in the Se/CS/AMP, the drug loading of Se/CS/AMP was measured (Fig. 9). The final drug loading of Se/CS/AMP’s loading was 23.8%.

### Antibacterial activity of Se NPs and se/CS/AMP

Growing bacterial resistance to commonly prescribed antibiotics has emerged as a significant global health concern in recent years. As a result, there is considerable interest in developing new antibacterial agents. In this study, Se NPs were produced using P. mirabilis PQ350419 and then combined with CS and AMP to boost their antibacterial effectiveness. The antibacterial properties of Se NPs and Se/CS/AMP were evaluated against various Gram-positive and Gram-negative bacteria, comparing them to the standard antibacterial AMP and CS through the agar well diffusion method, MIC, and MBC tests.

**Fig. 10:**
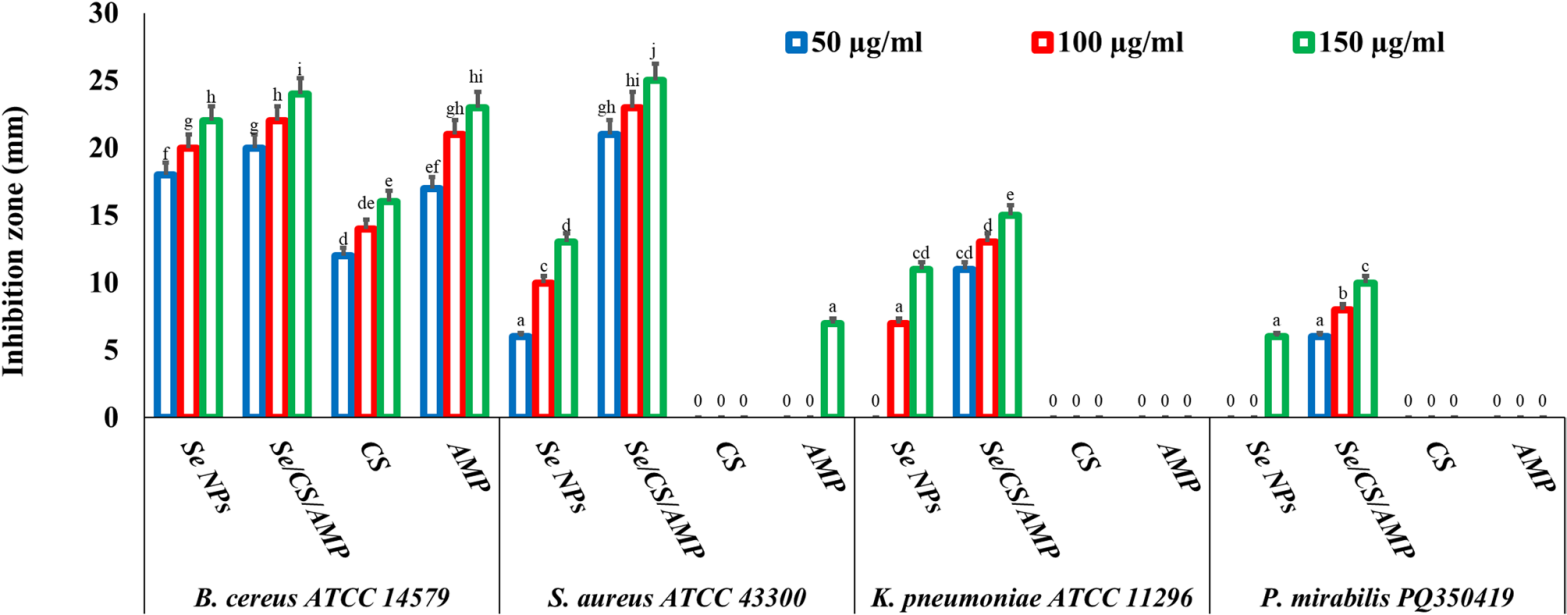
Antibacterial activity of Se NPs and Se/CS/AMP, CS, and AMP using agar well diffusion method against *B. cereus* ATCC 14,579, methicillin-resistant *S. aureus* ATCC 43,300, *K. pneumoniae* ATCC 11,296, and *P. mirabilis* PQ350419

**Fig. 11:**
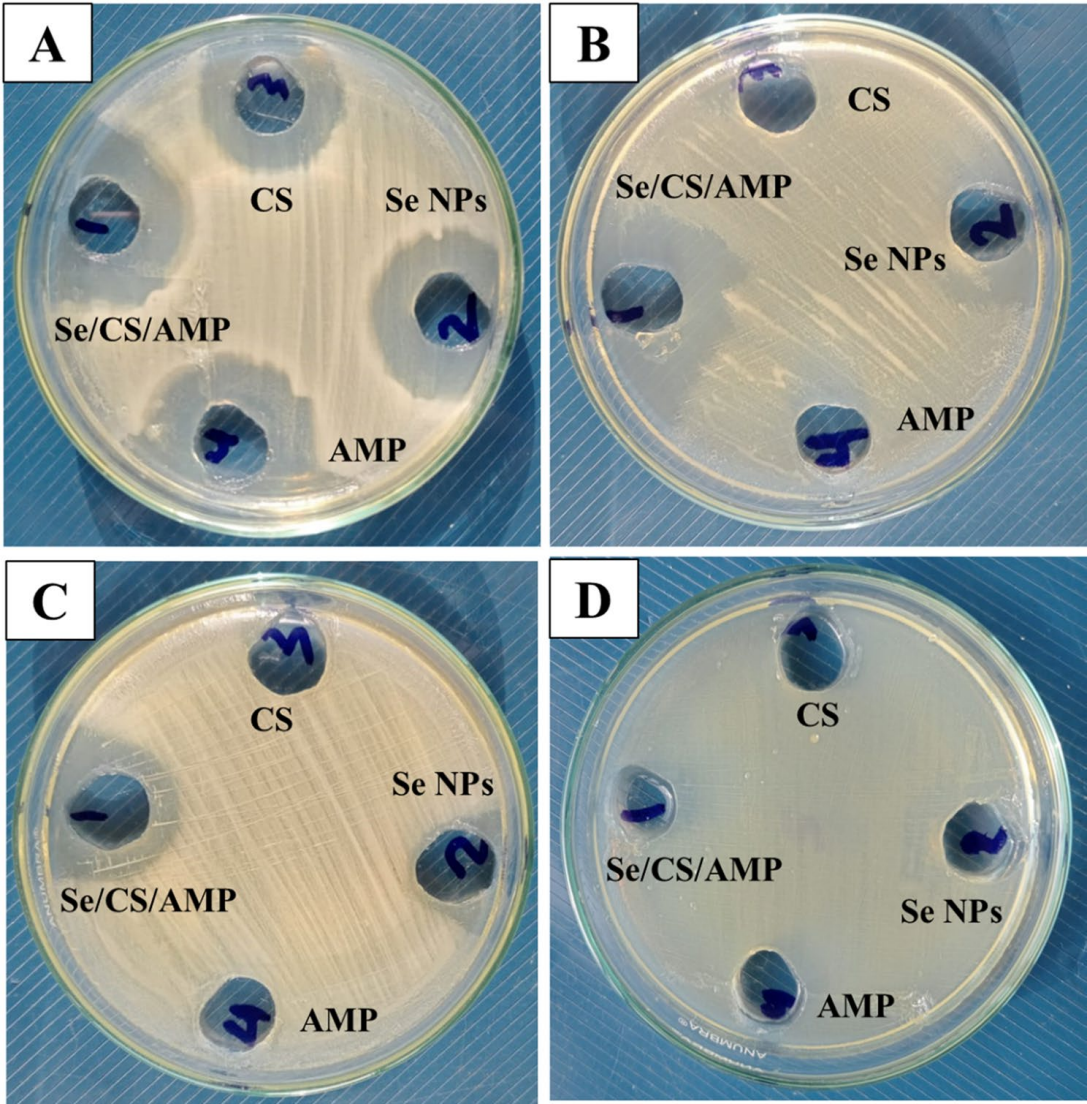
Agar well diffusion method test of Se NPs and Se/CS/AMP against *B. cereus* ATCC 14,579; (**A**), methicillin-resistant *S. aureus* ATCC 43,300; (**B**), *K. pneumoniae* ATCC 11,296; (**C**), and *P. mirabilis* PQ350419; (**D**), in comparison to AMP and CS

When an inhibition zone forms, bacteria are unable to proliferate, indicating that a treatment is effective against bacteria. The 150 µg/ml Se NPs led to the formation of inhibition zones of 22 ± 0.03 and 13 ± 0.06 mm against Gram-positive *B. cereus* ATCC 14,579 and *S. aureus* ATCC 43,300, as well as 11 ± 0.21 and 6 ± 0.14 mm against Gram-negative *K. pneumoniae* ATCC 11,296 and *P. mirabilis* PQ350419, respectively (Figs. 10 and 11). Se/CS/AMP showed superior antibacterial action compared to Se NPs, AMP, and CS against all tested bacteria. *K. pneumoniae* ATCC 11,296 and *P. mirabilis* PQ350419 displayed complete resistance to CS and AMP. The results obtained documented the resistance of the previous strains against the biosynthesized Se NPs with inhibition zones of 11 ± 0.19 and 6 ± 0.22 mm, respectively, compared to the prepared Se/CS/AMP, which had inhibition zones of 15 ± 0.14 and 10 ± 0.14 mm, respectively. In addition to the antibacterial potential of the biogenic Se NPs, their fold area increased by 0.17, 0.82, 0.72, and 0.88 against *B. cereus*, *S. aureus*, *K. pneumoniae*, and *P. mirabilis*. They also demonstrated significant synergistic effects when combined with CS and AMP. The potential use of Se NPs as effective single or combination antibacterial treatments in various environmental, industrial, and medical applications was confirmed by these results. Just as bacteria are known to develop resistance to antibiotics, they may also develop resistance to antimicrobial NPs. Nevertheless, NPs have been proven to be potent antimicrobials; multiple studies have demonstrated that bacteria have begun to develop resistance to NPs, including ZnO NPs and silver NPs [82, 83]. Efflux-mediated drug resistance in bacteria has been used to demonstrate how microbes have adapted to AMP or NPs alone. According to Niño-Martínez et al. [28], after repeated treatment and exposure to Ag NPs, *P. aeruginosa* CCM 3955, *E. coli* 013, and *E. coli* CCM 3954 develop resistance to the NPs. The presence of CS might enhance the adsorption force of the prepared Se/CS/AMP preventing and stopping the efflux action in drug-resistant bacteria and allowing Se/CS/AMP to have a full chance to act as a strong antibacterial agent.

It was noted that Se NPs and Se/CS/AMP are more efficient against Gram-positive than Gram-negative bacteria. Several variables, such as the type of bacteria, concentration of the antibacterial agent, surface area, as well as the size and shape of NPs, influenced the ZOI. Additionally, the structure and arrangement of the cell membrane may be associated with the variation in action against Gram-positive and Gram-negative bacteria [84]. The biosynthesized Se-NPs demonstrated greater efficiency against Gram-positive bacteria in our investigation. This is explained by a significant alteration in the composition of the bacterial walls. Gram-positive bacterial cell walls were found to have numerous holes, which enhance the entry of NPs to the bacterial cells, thereby increasing their interaction with bacterial components and enhancing their antibacterial action [85]. The Gram-negative bacterial cell wall has been described as having a multilayered cell wall, which could potentially prevent or decrease the entry rate of NPs into the bacterial cells [86]. Several studies have reported similar findings, suggesting that the synthesized Se NPs were more effective in combating Gram-positive bacteria than Gram-negative bacteria [78, 87].

**Fig. 12:**
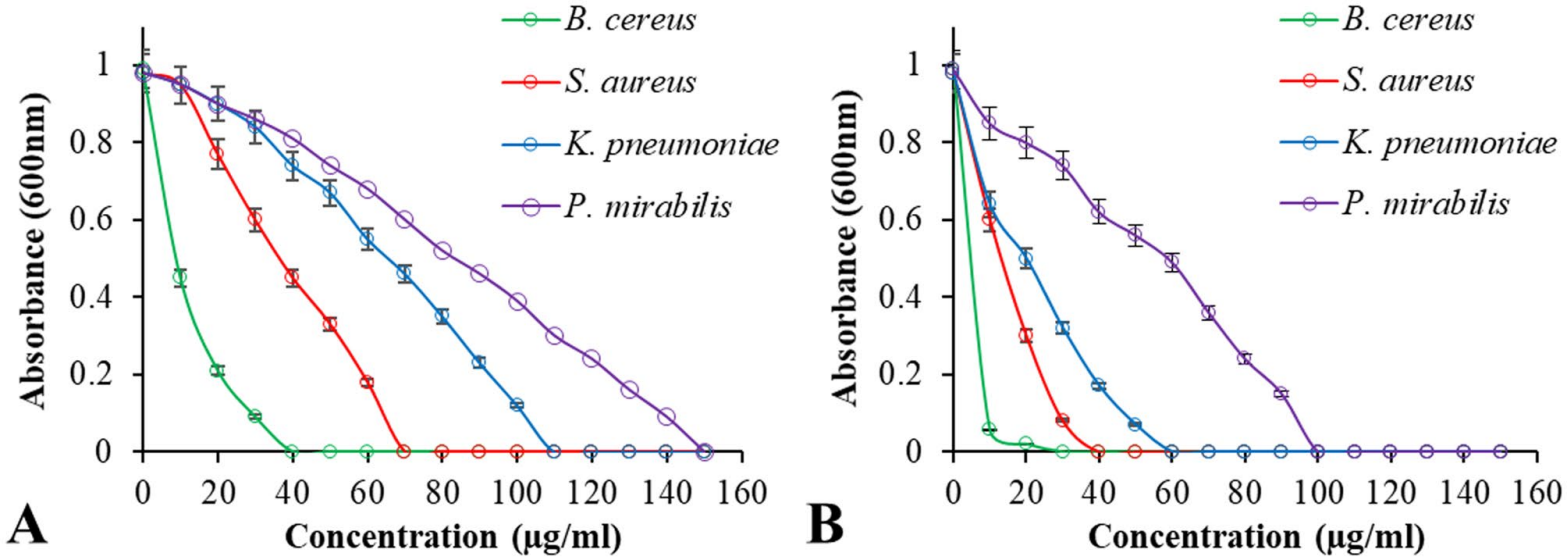
Minimum inhibition concentration of Se NPs; (**A**), and Se/CS/AMP; (**B**), against the tested bacteria

The antibacterial properties of the synthesized Se NPs and Se/CS/AMP were evaluated using MIC and MBC (Figs. 11 and 12). The MICs of Se NPs against *B. cereus* ATCC 14,579, *S. aureus* ATCC 43,300, *K. pneumoniae* ATCC 11,296, and *P. mirabilis* PQ350419 were 40 µg/ml, 70 µg/ml, 110 µg/ml, and 150 µg/ml, respectively. Meanwhile, the MICs of Se/CS/AMP were 30 µg/ml, 40 µg/ml, 60 µg/ml, and 100 µg/ml, respectively. The MBC values were 100 µg/ml and 30 µg/ml, respectively. This indicates that the biosynthesized Se NPs were more effective against Gram-positive bacteria than Gram-negative bacteria. Both Se NPs and Se/CS/AMP showed dose-dependent antibacterial activity against all test bacteria, which increased with their concentration. *P. mirabilis* PQ350419, followed by *K. pneumoniae* ATCC 11,296, showed less sensitivity to Se NPs compared to Se/CS/AMP. In order to demonstrate complete bacterial inhibition, higher concentrations of Se NPs (150 µg/ml) were required compared to the prepared Se/CS/AMP (100 µg/ml). When the MBC is no more than four times the MIC, an antibacterial agent is considered bactericidal [88]. The results for MBC are displayed, showing that the MIC values are consistent with the MBC values (Fig. 12). In contrast to the present findings, Charkhian et al. [89] reported high concentrations and MIC values for various NPs. For example, NiO NPs, Ag NPs, TiO_2_ NPs, and CuO had MIC values of 10,000, 2500, 15,000, and 1250 µg/ml, respectively, against *Proteus* sp. Furthermore, their MBCs were recorded as 15,000, 5000, 15,000, and 2500 µg/ml, respectively. The MIC and MBC of Se NPs against *B. cereus* BIPC04 were both found to be 75 µg/ml, as documented by Pouri et al. [90]. Additionally, while Se NPs had a MIC of 125 µg/ml against *B. cereus* ATCC 11,778 according to the study by Elgushi et al. [91]. Compared to the current results, biosynthesized Se NPs using *Lactobacillus acidophilus* revealed MIC values of 1–10 µg/ml against *E. coli*, *S. aureus*, and *B. subtilis.* In the case of drug-resistant bacteria such as *K. pneumoniae* and *P. aeruginosa*, the MICs were found to be 4 µg/ml and 6.5 µg/ml, respectively [23]. Khiralla & El-Deeb [92] reported the MIC values of biosynthesized Se NPs using the free-cell supernatant of *B. licheniformis* LN626649 against *B. cereus*, *S. aureus*, *E. coli* O157:H7, and *S. Enteritidis* as 40 µg/ml. In contrast, Se/CS demonstrated antibacterial efficacy against *S. aureus* NCIM 2079 with MIC and MBC values of 160 µg/ml and 320 µg/ml [93]. The biosynthesized Se NPs and Se/CS showed MICs against *S. aureus* and *S. typhimurium* at 6.25 µg/µl and 100 µg/µl, respectively, followed by *B. cereus* and *E. coli* at 12.5 µg/µl and 100 µg/µl [94].

### Ultrastructural study of Se NPs- and se/CS/AMP-treated *P. mirabilis* PQ350419

**Fig. 13:**
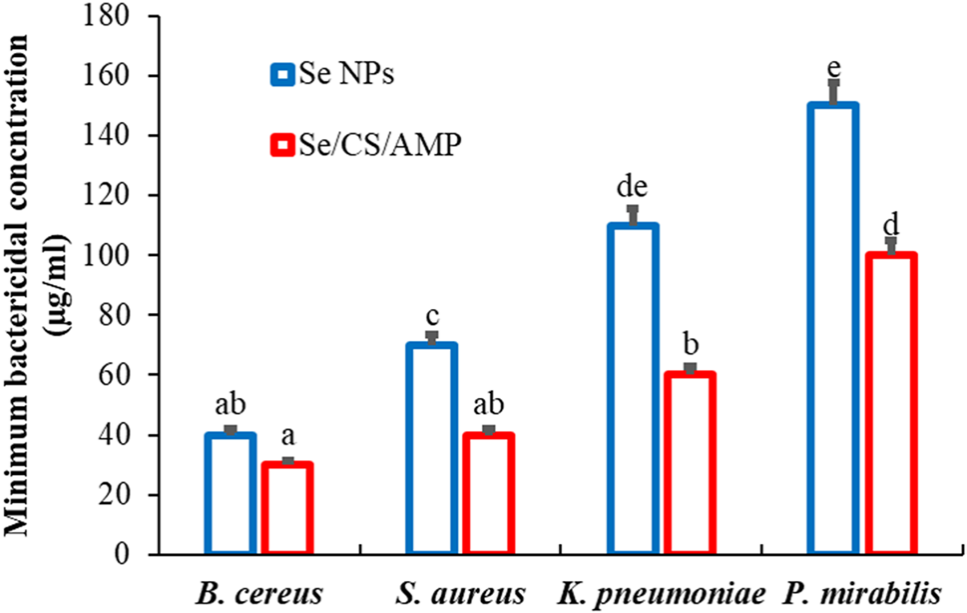
Minimum bactericidal concentration of Se NPs and Se/CS/AMP against the tested bacteria

**Fig. 14:**
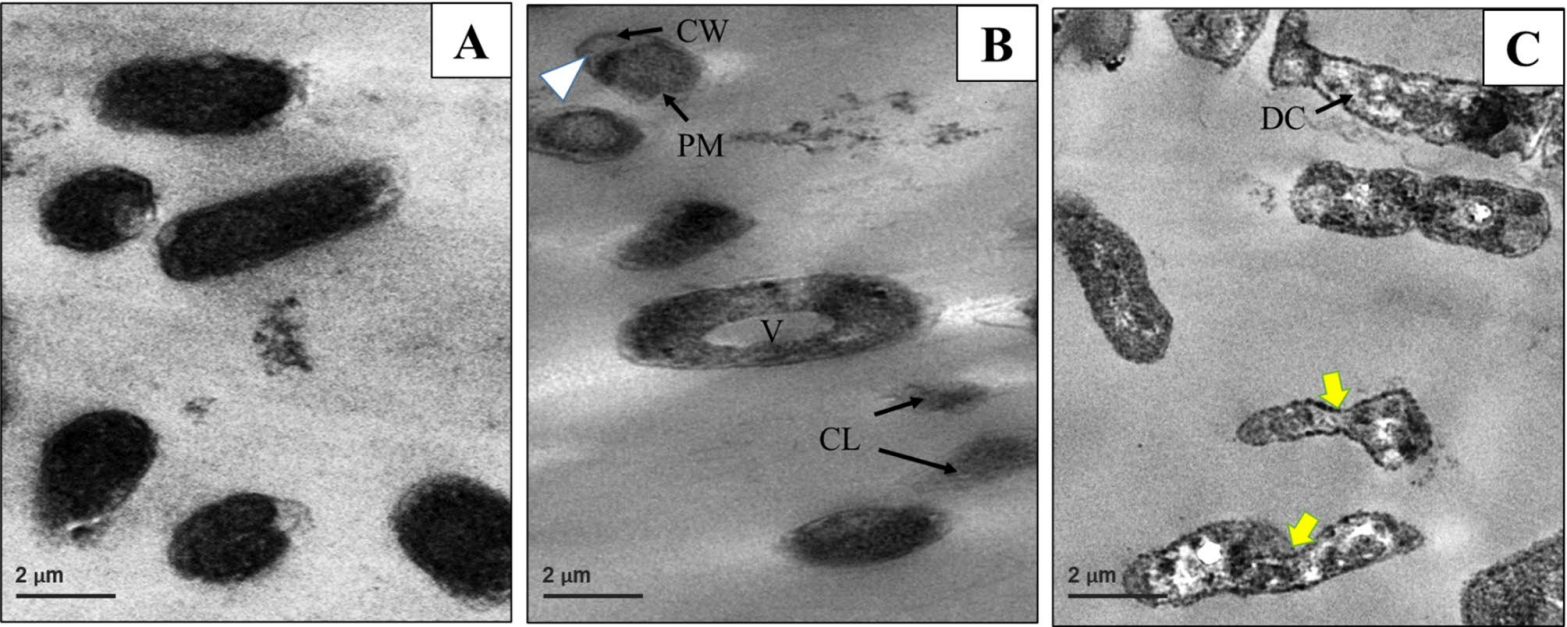
TEM micrographs showing the antibacterial action of Se NPs; (**B**), compared to Se/CS/AMP; (**C**) against *P. mirabilis* PQ350419. Untreated bacterial cells; (**A**), displayed normal cell structures. Treated *P. mirabilis* PQ350419 cells; (**B**&**C**), displayed separation between the cell wall (CW) and cytoplasmic membrane (PM); (white arrowhead), vacuole formation; (**V**), complete cell lysis; (CL), disintegrated cytoplasm (DC), and sever malformation and distortion; (yellow arrows)

A TEM micrograph was utilized to examine the antibacterial effects of Se NPs and Se/CS/AMP on *P. mirabilis* PQ350419 comparing them to untreated cells (Fig. 13). The untreated cells of *P. mirabilis* PQ350419 appeared to have a small vacuole, a homogenous and evenly dense cytoplasm, and a typical cell membrane and wall (Fig. 14A). In contrast, micrographs of *P. mirabilis* PQ350419 treated with Se NPs and Se/CS/AMP displayed several distinct morphological changes. These alterations included the cell wall rupturing, appearing wrinkled, the cell and cytoplasmic membranes clearly separating, and the development of a large vacuole (Fig. 14B&C). The outer membrane begins to exhibit blebbing and distortion, likely due to damage caused by nanomaterial exposure. The cytoplasm appears disorganized, suggesting stress or damage. Furthermore, the periplasmic space may be enlarged due to the breakdown of the bacterial cell wall and loss of cellular contents. When the cell wall and membrane deteriorate, the cell may collapse or exhibit signs of autolysis. Various TEM investigations and micrographs of bacterial cells treated with Se NPs and Se NCs [95–98] showed significant changes in the bacterial cells’ contents, including damaged cell walls and extensive leakage of cytoplasmic matrix.

All recent bacterial micrographs treated with Se NPs and Se NCs showed that these nanomaterials had strong bactericidal effects on the cell membranes and cell walls. This resulted in the appearance of extensively damaged cells, with the autolysis of the majority of cell organelles occurring. The mechanisms of Se NPs are also linked to the creation of ROS, the interaction with the cell barrier (cell-wall rupture and permeability alteration), the suppression of protein and DNA synthesis, metabolic gene expression, and other factors, as reported in several studies [99]. The production of ROS by metal-based NPs (superoxide anions, hydroxyl radicals, and hydrogen peroxide) is often associated with the antibacterial effect. According to several studies, Se NPs can generate ROS [100, 101]. Future research should examine these significant effects, as these types of ROS may further inhibit the replication of DNA and amino acids, as well as damage bacterial cell membranes [102].

### Antibiofilm potential of Se NPs and se/CS/AMP

**Fig. 15:**
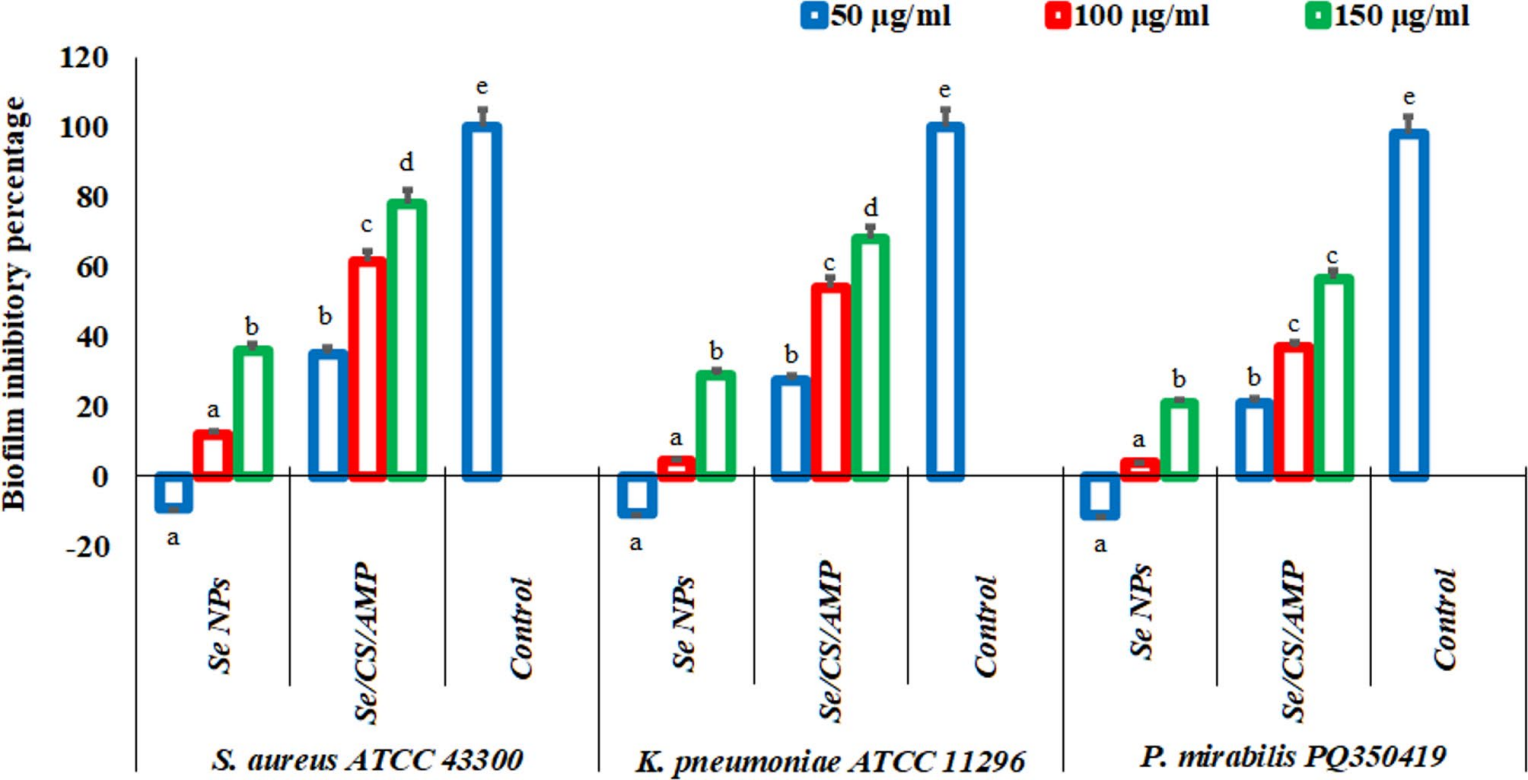
Antibiofilm potential of Se NPs and Se/CS/AMP in different concentrations (50, 100, and 150 µg/ml) against biofilm-producing bacteria (*S. aureus* ATCC 43300, *K. pneumoniae* ATCC 11296, and *P. mirabilis* PQ350419)

The antagonistic potency of Se NPs and Se/CS/AMP in inhibiting of biofilm formation by biofilm-producing bacterial strains, including *S. aureus* ATCC 43,300, *K. pneumoniae* ATCC 11,296, and *P. mirabilis* PQ350419, was investigated (Fig. 15). The tested bacteria showed higher resistance to Se NPs compared to Se/CS/AMP. As the concentration of Se NPs and Se/CS/AMP increased, biofilm inhibition also increased. All the bacterial strains investigated showed a significant inhibition of biofilm formation that increased with concentration. The prepared nanomaterials were found to be more effective against *S. aureus* compared to *K. pneumoniae* and *P. mirabilis.*

According to similar findings by Liu et al. [103] and Slany et al. [104], the application of sub-inhibitory concentrations of methicillin or any other disinfectant can significantly induce the formation of *S. aureus* biofilms by up-regulating the genes encoding surface proteins involved in the biofilm formation process. Furthermore, instead of inhibiting the production of biofilms, the essential microelement Se stimulates the biofilm-forming microorganisms. In our work, bacteria used Se at its lowest concentration (50 µg/ml) to regulate physiological processes and numerous metabolic activities. Similarly, Khiralla & El-Deeb [92] reported that the biosynthesized Se NPs did not show the ability to remove the established biofilm lower than 50 µg/ml against *B. cereus*, *S. aureus*, *E. coli* O157:H7, and *S. Enteritidis*. The findings byAbu-Elghait et al. [105] show that Se NPs strongly inhibited the biofilm development of *P. aeruginosa* and MRSA up to 86.4% at 18.7 and 150 ppm, respectively, while Se NCs suppressed their biofilm formation up to 22.9% at 150 ppm. In contrast, Miglani & Tani-Ishii [106] highlighted that 1000 µg/ml of Se NPs were required to stop the development and make the eradication process of bacterial biofilm formation. Further research needed to investigate the mechanism of action for Se NPs and Se/CS/AMP in destroying biofilms producing by bacteria.

### Cytotoxicity assay

**Fig. 16:**
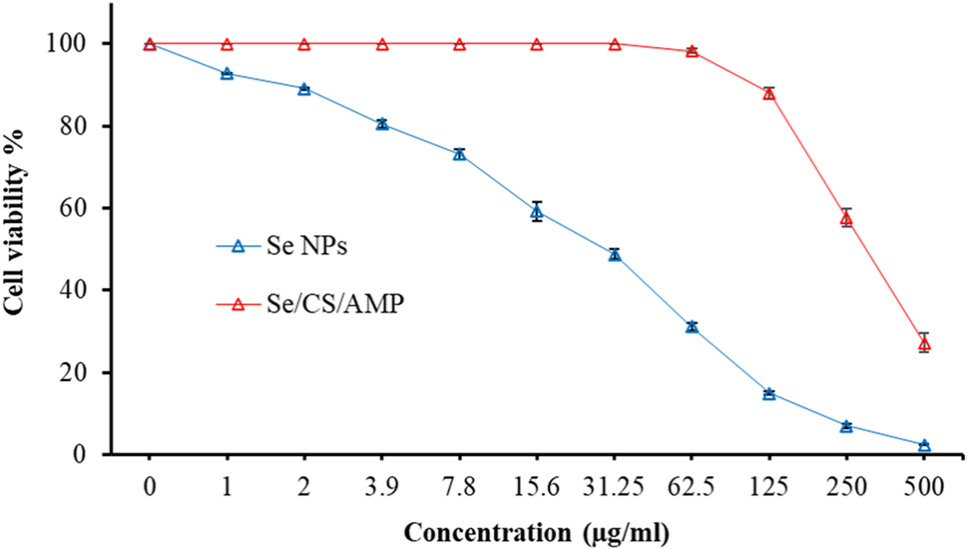
The cytotoxic effects of Se NPs and Se/CS/AMP against Vero cell line

Chemically produced NPs were found to be both cytotoxic and genotoxic, while NPs produced through green methods did not have any harmful or fatal effects on normal cell lines [107, 108]. Consequently, Vero cells are used to test and evaluate the toxicity of the produced Se NPs and Se/CS/AMP (Fig. 16). The relative CC_50_ values for Se NPs and Se/CS/AMP were 40.95 ± 2.34 and 199.09 ± 2.61 µg/ml, respectively. This outcome validates the safety of using green prepared Se/CS/AMP at lower concentrations, including MICs (30–100 µg/ml). A CC_50_ of 165.5 ± 5.4 µg/ml was found when Se NPs were examined against Vero cells as reported by Fouda et al. [109]. Anu et al. [110] discovered that biologically synthesized Se NPs had a lower cytotoxicity a more environmentally friendly CC_50_ (31.8 ± 0.6 µg/ml) compared to chemically manufactured Se NPs (18.8 ± 0.8 µg/ml), According to Mohammed et al. [111] and Mohamed & El-Zahed [12], Vero cells exhibited 100% viability at 41.5 µg/ml and 31.25 µg/ml of Se, respectively, but viability declined as Se NP concentrations increased. The ZnO/Se nanocomposite exhibited a CC_50_ of 162.02 ± 3.14 µg/ml, according to Fayed et al. [65].

## Conclusions

The bacterial isolate *P. mirabilis* was identified using the Vitek 2 and 16S rRNA gene sequence and added to the database with accession number PQ350419. This strain was used to fabricate and optimize the production of Se NPs extracellularly as a simple green approach. The biogenic Se NPs were combined with chitosan to produce the final nanocomposite known as Se/CS/AMP. Zeta potential analysis, FTIR, XRD, TEM, and UV-Vis spectroscopy were utilized to verify the synthesis of Se NPs and Se/CS/AMP. The presence of peaks at 3000–2800 cm^− 1^ in the zeta potential and FTIR spectrum of the Se NPs and Se/CS/AMP could act as stabilizing agents, indicating the long-term stability of the particles. The data obtained from the antibacterial studies including the agar well diffusion method, MIC, and MBC indicate that Gram-positive bacteria are more susceptible to Se/CS/AMP than Gram-negative bacteria. MIC values for the green- synthesized nanomaterials ranged from 40 to 150 µg/ml for Se NPs and from 30 to 100 µg/ml for Se/CS/AMP, indicating strong bactericidal action against both Gram-positive and Gram-negative bacteria. Additionally, both Se NPs and Se/CS/AMP were able to inhibit the biofilm formation ability of *S. aureus* ATCC 43,300, *K. pneumoniae* ATCC 11,296, and *P. mirabilis* PQ350419. The current study found that Se/CS/AMP displayed a low toxic effect on Vero cells, with a CC_50_ = 199.09 ± 2.61 µg/ml compared to Se NPs (40.95 ± 2.34 µg/ml). Interestingly, the mutual cooperative interactions of Se NPs and Se/CS/AMP are of great interest for potential medical and pharmaceutical applications. Future research could further explore the mode of action of antibacterial, antibiofilm, and toxicity of Se/CS/AMP using in vivo studies on an animal model.

## Data Availability

The 16S ribosomal RNA gene partial sequence of Proteus mirabilis strain AUF1 acquired during the current investigation has been added to the NCBI GenBank database with accession number PQ350419 and can be accessed at https://www.ncbi.nlm.nih.gov/nuccore/PQ350419.1. Upon reasonable request, the corresponding author will provide the datasets generated during the current work. Upon reasonable request, the relevant author will make the datasets used and/or analyzed in the current work available.
